# Editorial: Gut biodiversity and its influence in brain health

**DOI:** 10.3389/fnins.2023.1221543

**Published:** 2023-06-01

**Authors:** Silvia Turroni, Gustavo Provensi

**Affiliations:** ^1^Unit of Microbiome Science and Biotechnology, Department of Pharmacy and Biotechnology, University of Bologna, Bologna, Italy; ^2^Department of Neuroscience, Psychology, Drug Research and Child Health (NEUROFARBA), Section of Pharmacology of Toxicology, University of Florence, Florence, Italy

**Keywords:** brain, alpha diversity, necrotizing enterocolitis, gut microbiota, neurodegenerative diseases

The research fields of neuroscience and gut microbiota have essentially evolved along distinct trajectories. Even though these independent roads have crossed a few times in the last century, most researchers point to the demonstration that the commensal microbiota can affect the neural network responsible for controlling stress responsiveness (Sudo et al., 2004) as the seminal paper for the microbiome-gut-brain field. The gut-brain axis goes far beyond this concept and involves a close interaction between gut microbes and brain health, mediated by the production of neuroactive microbial products, modulation of hormone secretion and immune system, and direct stimulation of afferent fibers, including the vagus nerve (Shoubridge et al., 2022). While there is still a long way to go, microbiota alterations have been associated with the onset and progression of some neurological disorders (Mitrea et al., 2022), with tantalizing promise for identifying diagnostic biomarkers and developing new therapeutic approaches.

To integrate the available knowledge and further promote the development of the field, we proposed a Research Topic entitled “*Gut biodiversity and its influence in brain health*,” for collecting up-to-date manuscripts on the impact of gut dysbiosis on brain health.

In a bibliometric analysis, Yang et al. generated knowledge maps from gastrointestinal microbiome and neuroscience studies published in the Web of Science Core Collection database. A total of 2,275 articles, including 1,433 original research articles and 842 reviews, were published in 2002–2022, with a sharp increase since 2016. The United States and China have emerged as the most notable contributors to gastrointestinal microbiome and neuroscience research, although Prof. John Cryan from Ireland is recognized as the most productive and cited author, having played a leading role in the field. Co-occurring keyword analysis revealed the gastrointestinal microbiome, inflammation, gut-brain axis, Parkinson's disease, and Alzheimer's disease as major hotspots over the past two decades and suggests research on “gastrointestinal microbiome, inflammation: the link between obesity, insulin-resistance and cognition” and “the role of two important theories of the gut-brain axis and microbial-gut-brain axis in diseases in neuroscience” as future hotspots.

In line with this, two out of five papers on our Research Topic focused on understanding the link between gut dysbiosis and neurodegenerative diseases. In a systematic review and meta-analysis, Li et al. compared six α-diversity indices, including community richness (observed species, Chao1 and ACE), community diversity (Shannon, Simpson), and phylogenetic diversity, between controls and patients suffering from different brain-related disorders across the world. No differences between patients and controls emerged in the overall analysis. However, subgroup analyses revealed that α-diversity indices were significantly altered in anorexia nervosa and Parkinson's disease (PD) patients, being increased in the former and reduced in the latter. Furthermore, receiver operating characteristic curve analysis showed that gut dysbiosis measured as α-diversity could be a promising predictor for Alzheimer's disease (AD), schizophrenia, and multiple sclerosis (MS). In the second study, Chauhan et al. performed an *in-silico* comparative analysis of microbial composition and metabolic pathway alterations occurring in neurodegenerative diseases, including AD, PD, MS, and amyotrophic lateral sclerosis (ALS). Interestingly, the authors found a striking similarity in dysbiotic signatures between AD, PD, and MS, while ALS had a slightly different microbial profile. Accordingly, similarities in microbial populations were observed: overrepresented microbes belonged to the phyla Bacteroidetes, Actinobacteria, Proteobacteria, and Firmicutes, while underrepresented ones mainly belonged to Bacteroidetes and Firmicutes. Using the MACADAM (MetAboliC Pathways Database for Microbia taxonomic groups) tool, the possible metabolic profile resulting from dysbiosis was identified. Highly prevalent microbes lacked pathways for synthesizing the short-chain fatty acids acetate and butyrate. Also, these microbes had increased ability to produce L-glutamate, an excitatory neurotransmitter and precursor of GABA. Contrastingly, tryptophan (precursor of serotonin), histamine and spermidine (a neuroprotective polyamine) synthetic machinery were less represented. These results may pave the way for the development of microbiome-based diagnostic and therapeutic approaches.

Despite improvements in neonatal intensive care, mortality rates from necrotizing enterocolitis (NEC), the most common life-threatening emergency affecting the gastrointestinal tract of preterm infants, are still high (30–50%). NEC survivors present several damages, including neurodevelopmental delay and severe neurocognitive impairments. The exact pathogenetic mechanisms of brain injury in NEC remains unknown, but severity appears to be related to the extent of neuroinflammation. Wang et al. addressed a very interesting hypothesis of the microbiota-gut-brain axis contribution to NEC pathophysiology in preterm infants. The idea is that NEC is associated with inappropriate intestinal colonization and consequential dysbiosis. Some studies have in fact demonstrated the efficacy of probiotics in reducing the risk of NEC or severe NEC by regulating the gut microbiome. These studies have some limitations, particularly regarding their limited size. Further investigations, also focusing on the metabolome, could help identify biomarkers to accurately and timely predict NEC and select appropriate and effective treatments.

Finally, the review by Deidda and Biazzo highlights the role of bidirectional microbiota-gut-brain communication in physiological processes, as well as the alterations observed in brain-related pathologies, i.e., neurodevelopmental disorders (Autism Spectrum Disorder, Schizophrenia, Rett and Down Syndromes), neurodegenerative diseases (AD and PD) and mood disorders (depression). It is impressive how diseases showing completely different clinical manifestations share a common gut dysbiosis. It is necessary to recognize, however, that it is often unclear if disease-associated microbiota changes are meaningful, and the distinction between cause and effect is inherently challenging. Despite this, the alterations observed may offer valid possibilities for the development of innovative therapies. In fact, the last part of the review presents and discusses the studies evaluating the efficacy of pre/probiotics in the above-mentioned disorders. The results are quite promising, but many pieces are missing to solve the puzzle: regulatory issues, limited choice of bacteria available, survival of the bacteria after stomach transit, effective gut colonization ad so on.

In summary, we believe that the contributions gathered in this Research Topic provide an excellent overview of the state of the art of research in gut microbiome and brain health, highlighting current promises and limitations, and pointing the way forward. In particular, the current results suggest the relevance of certain taxa and metabolites of the gut microbiome as diagnostic biomarkers and/or therapeutic targets in neurological disorders. However, it must be said that studies are still few, very often in small cohorts, and mostly of an associative nature. Other omics approaches, including metabolomics, and animal models should be used to unravel the underlying mechanisms of action. Once this knowledge is achieved, it will finally be possible to design microbiome-based intervention strategies to tackle specific dysbiotic states and promote brain health.

## Author contributions

GP: original draft preparation. GP and ST: review and editing. Both authors have read and agreed to the published version of the manuscript.
